# Sepsis-induced endothelial glycocalyx disruption and remodeling: an ultrastructural study in mice

**DOI:** 10.1186/s40635-026-00954-w

**Published:** 2026-07-20

**Authors:** Ryo Hisamune, Kazuma Yamakawa, Hong Wu, Yoshihiko Fujioka, Katsuhide Kayano, Noritaka Ushio, Rintaro Oide, Masahiro Terasawa, Koji Suzuki, Takashi Nakano, Akira Takasu

**Affiliations:** 1 Department of Emergency and Critical Care Medicine, Osaka Medical and Pharmaceutical University, 2-7 Daigakumachi, Takatsuki, 569-8686 Osaka Japan; 2 Department of Microbiology and Infection Control, Faculty of Medicine, Osaka Medical and Pharmaceutical University, Takatsuki, Japan; 3 Department of Translational Research, Osaka Medical and Pharmaceutical University, Takatsuki, Japan; 4 Faculty of Pharmaceutical Sciences, Suzuka University of Medical Science, Suzuka, Japan; 5 Konan Chemical Manufacturing, Co., Ltd, Yokkaichi, Japan

**Keywords:** Sepsis, Glycocalyx, Rhamnan sulfate, Inflammation, Electron microscopy

## Abstract

**Background:**

Sepsis damages endothelial cells through glycocalyx degradation, contributing to organ dysfunction. Glycocalyx repair is essential for recovery, but the underlying mechanisms remain unclear. Rhamnan sulfate (RS), a sulfated polysaccharide with anti-inflammatory properties, may support endothelial glycocalyx repair. In this study, we investigated the glycocalyx repair process and the therapeutic potential of RS using a mouse sepsis model.

**Methods:**

Sepsis was induced in male C57BL/6 mice by cecal ligation and puncture (CLP). Mice were divided into the RS, control (CTL), and sham groups. RS was orally administered to the RS group, while the CTL group received no treatment after CLP. Sham mice underwent laparotomy only without CLP. Endothelial glycocalyx repair was evaluated using transmission electron microscopy (TEM) and scanning electron microscopy (SEM), and quantitative TEM analysis. Gene expressions of glycocalyx-related enzymes and inflammatory cytokines were assessed in liver and small intestine tissues by quantitative real-time PCR.

**Results:**

The 7-day survival rate after CLP was significantly higher in the RS group (55%) than in the CTL group (25%). TEM and SEM demonstrated that in the CTL group, the endothelial glycocalyx remained disrupted for up to 72 h after CLP but by two weeks had undergone compensatory regeneration with thickening, exceeding that observed in sham-treated mice. In contrast, in the RS group, glycocalyx continuity was restored by 72 h and normalized to levels comparable to those of the sham-treated group by two weeks. Quantitative TEM analysis supported marked structural alteration of glycocalyx after CLP and normalization of glycocalyx thickness toward sham levels after RS treatment. Quantitative real-time PCR revealed a significant increase in inflammatory cytokines (IL-1β, IL-6, TNF-α) after CLP, which tended to be lower in the RS-treated group. A similar trend was observed for the glycocalyx-degrading enzymes heparanase and hyaluronidase, whose expression was also suppressed by RS treatment.

**Conclusions:**

Electron microscopy allowed detailed observation of endothelial glycocalyx repair in septic mice. RS promoted glycocalyx repair and reduced inflammation, suggesting that administration of RS may be a novel therapeutic strategy for maintaining vascular integrity in sepsis.

**Supplementary Information:**

The online version contains supplementary material available at 10.1186/s40635-026-00954-w.

## Background

Sepsis is a life-threatening disease caused by an excessive immune response to an invading microbial infection [1, 2]. Despite advances in clinical treatment, mortality remains high at 20–40% [3, 4]. Severe sepsis often causes vascular endothelial damage and leads to organ dysfunction. The vascular endothelium is protected by the glycocalyx, a complex layer of polysaccharides and glycoproteins [5]. However, its structure deteriorates during sepsis [6, 7]. This damage increases vascular permeability, impairs microcirculation, and contributes to organ dysfunction. Notably, the structure of glycocalyx varies between organs, and the pattern of degradation differs depending on the type of inflammation [8]. During regeneration, the glycocalyx is remodeled by the incorporation of both endothelium-derived components and circulating plasma components into the meshwork [9]. Previous studies have shown that exogenous supplementation with glycocalyx components (e.g., chondroitin sulfate and hyaluronic acid) promotes the reconstruction of damaged glycocalyx layers [10]. Although various therapeutic agents have been investigated to promote glycocalyx repair, none have yet been put into clinical practice [11, 12]. Therefore, despite great interest, our understanding of the glycocalyx repair process remains limited. Elucidating the mechanism of glycocalyx repair in sepsis and identifying factors that promote its recovery are essential for developing effective therapeutic strategies.

In recent years, rhamnan sulfate (RS) has emerged as a potential regulator for glycocalyx maintenance and repair [13]. RS is a soluble dietary fiber in the intercellular substances, composed of linear and branched rhamnose units, approximately 25% of which contain sulfate groups [14, 15]. This sulfated polysaccharide, derived from the green alga *Monostroma nitidum*, has demonstrated anti-inflammatory properties [16]. Specifically, oral administration of RS has been shown to modulate the composition of gut microbiota, enhance intestinal function, and contribute to the regulation of systemic inflammation [16, 17]. Our previous studies have shown that orally administered RS protects the vascular endothelium under inflammatory conditions. In a lipopolysaccharide‑induced acute inflammation model, prophylactic RS reduced vascular leakage, attenuated organ injury and inflammatory responses, and preserved the pulmonary endothelial glycocalyx [16]. In a separate study using ApoE‑deficient mice, RS ameliorated dyslipidemia and reduced vascular inflammation, indicating vasculoprotective effects also in chronic inflammatory settings [18]. However, whether RS can promote glycocalyx recovery after the onset of sepsis remains unknown. The present study was therefore designed to evaluate the therapeutic effect of post‑insult oral RS administration in a cecal ligation and puncture (CLP) model, with a particular focus on time‑dependent ultrastructural injury and repair of the endothelial glycocalyx.

This study aimed to evaluate the repair process after sepsis-induced endothelial cell glycocalyx detachment and to elucidate the underlying mechanisms and regulatory factors using a mouse model of sepsis. The repair process was assessed using electron microscopy, and key factors involved in the repair were identified by analyzing the mRNA expression levels of glycocalyx-related enzymes (glucuronyltransferase [GlcAT], heparanase [HPSE], and hyaluronidase [HYAL2]) and inflammatory cytokines (interleukin-1β [IL-1β], interleukin-6 [IL-6], tumor necrosis factor-α [TNF-α], and transforming growth factor-β [TGF-β]). Furthermore, we examined the effects of oral RS administration given its potential glycocalyx-protective properties.

## Methods

### Animals

Six-week-old male C57BL6 mice were purchased from Japan SLC, Inc. (Hamamatsu, Shizuoka, Japan) and acclimated to the experimental environment for two weeks. All mice were housed in our specific pathogen-free facility under controlled temperature and humidity conditions, maintained on a 12-hour light/dark cycle, with *ad libitum* access to standard chow and water. This study adhered to the guidelines outlined in the Guide for the Care and Use of Laboratory Animals, published by the National Institutes of Health (NIH, 8th Edition, 2011), and received approval from the Institutional Animal Research Committee at Osaka Medical and Pharmaceutical University in Osaka, Japan (approval numbers: MA24-013 and MA26-031).

### Study design

This exploratory in vivo study evaluated the effects of RS in a mouse sepsis model. Mice were assigned to sham, control (CTL), or RS groups. Other than using healthy six‑week‑old male C57BL/6 mice, no predefined inclusion or exclusion criteria were applied, and no animals or samples were excluded. Randomization was not performed, and procedures were conducted in a fixed order. Blinding was not feasible because treatment and sample processing differed between groups. Multiple outcomes were assessed, including survival, endothelial glycocalyx morphology, and gene expression profiles.

This study was designed as an exploratory pilot investigation. Because no prior in vivo data were available to estimate the effect size of RS treatment in sepsis, a formal a priori sample size calculation was not performed. Animal numbers were minimized in accordance with the ARRIVE guidelines and principles of animal welfare [19]. No post-operative analgesia was administered because it could confound the evaluation of inflammatory responses and endothelial glycocalyx remodeling. Animals were monitored daily, and predefined humane endpoints were strictly applied.

### Preparation of RS

RS was obtained from a hot-water extract of *Monostroma nitidum* and purified following a previously reported procedure [13]. The purified material (94% purity; Konan Chemical Manufacturing Co., Ltd., Yokkaichi, Mie, Japan) displayed a single principal peak with a small leading shoulder on gel-permeation chromatography, with an average molecular weight of 5 × 10^5^ Da.

### Experimental procedure

Following acclimation, mice were anesthetized intraperitoneally with a combination of medetomidine (0.3 mg/kg), midazolam (4.0 mg/kg), and butorphanol (5.0 mg/kg). CLP was performed to induce sepsis, and 1.2 mL of saline was injected subcutaneously to support hydration. CLP was induced by ligating the cecum 1 cm from the apex and creating a single puncture 5 mm from the apex with a 26-gauge needle. The expected survival rate at one week after CLP induction was 30%.

In the RS group, RS (10 mg) was administered orally at 24 h and 48 h after CLP, followed by a daily oral dose of 5 mg until day 14. In the CTL group, mice underwent the CLP procedure but received standard housing conditions without the administration of RS. Sham mice underwent laparotomy alone without CLP.

### Survival analysis

Mice in the sham, CTL, and RS groups were monitored for 7 days to determine survival (*n* = 6, 20, and 20, respectively). Mortality was assessed once daily throughout the 7‑day observation period.

### Electron microscopy

Electron microscopy was performed on the liver and kidney, which are standard vascular beds for assessing glycocalyx ultrastructure in sepsis. Liver samples were obtained from the median lobe, and analyses were performed on hepatic sinusoidal endothelial cells. Kidney samples were collected from the peripheral cortical region, and electron microscopy focused on glomerular capillary endothelial cells. These vascular compartments were selected based on their well‑characterized endothelial phenotypes [20]. Ultrastructural evaluation of the intestinal microvasculature was not feasible due to tissue fragility and the complex villus–crypt architecture. Electron microscopic examination of the endothelial glycocalyx in the liver and kidney was performed following a previously established method [21]. Briefly, mice were anesthetized with a triple anesthesia cocktail and perfused through a cannula inserted into the left ventricle at a constant flow rate of 2 mL/min using a perfusion solution containing 2% glutaraldehyde, 2% sucrose, 0.1 M sodium cacodylate buffer (pH 7.3), and 2% lanthanum nitrate. After perfusion fixation, the liver and kidneys were sectioned into 5-mm squares for scanning electron microscopy (SEM) and 1-mm cubes for transmission electron microscopy (TEM) analysis. The liver and kidney samples were fixed in the perfusion solution for 2 h, then transferred to glutaraldehyde-free solution at 4 °C overnight. After that, these samples subsequently were washed with an alkaline (0.03 M NaOH) 2% sucrose solution.

For SEM, samples were prepared using the freeze-fracture method. All of the sections were observed by SEM (TM-4000PlusII, Hitachi, Tokyo, Japan). For TEM, the samples were dehydrated in ethanol solutions of serially graded concentrations and embedded in epoxy resin (Nisshin EM, Tokyo, Japan). Ultrathin sections were prepared using a Power Tome X ultramicrotome (RMC Boeckeler Instruments, Inc., Tucson, AZ, USA) and mounted on a copper grid (200 mesh). The ultrathin sections were subjected to conventional double staining with 3% (w/v) uranyl acetate for 10 min and with Reynolds’ Pb for 3 min for TEM. All the sections were observed by TEM (HT7800 types, Hitachi, Tokyo, Japan). Digital electron micrographs were captured with a CCD camera (XR-81) at 5,000 − 15,000× magnification. The pixel sizes of the micrographs were 322 ppi and 3296 × 2472. SEM and TEM were both conducted at 24 h, 72 h, and two weeks after CLP.

Quantitative analysis of the endothelial glycocalyx structure was performed using TEM. For glycocalyx thickness, we randomly selected ≥ 100 perpendicular measurement points per group from multiple TEM sections and measured the vertical height of the electron‑dense glycocalyx layer. All measurements were performed using ImageJ, and mean values per animal were used for statistical analysis. For linear coverage, endothelial segments of 10 μm were used as a standardized measurement unit. From multiple TEM sections per animal, we obtained ≥ 25 measurement points per group and calculated the proportion of the luminal endothelial surface covered by identifiable electron‑dense glycocalyx.

### Quantitative real-time PCR (qPCR)

Liver and small intestine tissues were collected from the sham mice (without CLP) and from the CTL and RS groups, at 48 h after CLP induction (*n* = 4 per group). The small intestine was included because orally administered RS is expected to exert primary effects at the intestinal interface, including absorption and modulation of local inflammation. The kidney was not used for qPCR because RNA yield and target gene expression changes were insufficient for reliable quantification. In the RS group, 10 mg of RS was administered orally 24 h after CLP. After anesthesia with a triple cocktail, liver and small intestine tissues were collected and immersed in RNAlater Stabilization Solution (Invitrogen, Waltham, MA, USA). Samples were stored at 4 °C overnight to preserve RNA integrity. Total RNA was extracted using an RNeasy Mini Kit (QIAGEN, Hilden, Germany), and automated extraction was performed using the QIACUBE Connect (QIAGEN). RNA concentration and purity were assessed using a NanoDrop spectrophotometer (Thermo Fisher Scientific). Complementary DNA was synthesized from 500 ng of total RNA using SuperScript IV VILO Master Mix (Thermo Fisher Scientific). qPCR was conducted using TaqPath qPCR PCR Master Mix, CG (Applied Biosystems) on a QuantStudio 5 Real-Time PCR System (Applied Biosystems). The mRNA expression levels of GlcAT, HPSE, HYAL2, IL-1β, IL-6, TNF-α, and TGF-β were analyzed. Relative expression levels were calculated using the 2^−ΔΔCt^ method with GAPDH as the internal control.

### Plasma biomarker and cytokine measurements

Plasma biomarkers of glycocalyx shedding and cytokine were quantified in six groups (Sham, CLP‑24 H, CLP‑48 H, CLP‑72 H, CLP‑48 H + RS, CLP‑72 H + RS) using samples collected from 4 to 6 animals per group. Plasma syndecan‑1 was measured using a murine CD138 ELISA kit (Cat# 860.090.096; Diaclone SAS, Besançon, France), and plasma hyaluronan was measured using a Quantikine Hyaluronan Immunoassay (Cat# DHYAL0; Bio‑Techne/R&D Systems). Plasma cytokines (IL‑1β, IL‑6, and TNF‑α) were quantified using a custom ProcartaPlex Multiplex Panel (Thermo Fisher Scientific) according to the manufacturer’s instructions.

### Statistical analysis

Survival analysis was performed using the log‑rank test. Group differences in mRNA expression, plasma glycocalyx biomarkers, and plasma cytokines were analyzed using the Kruskal-Wallis test followed by Dunn’s multiple‑comparisons test. Only pre‑specified biologically relevant pairwise comparisons are shown in the figures. P values < 0.05 were considered statistically significant. All statistical analyses were performed using Prism (ver. 10.2.3, GraphPad, La Jolla, CA, USA).

## Results

### Survival analysis

**Fig. 1:**
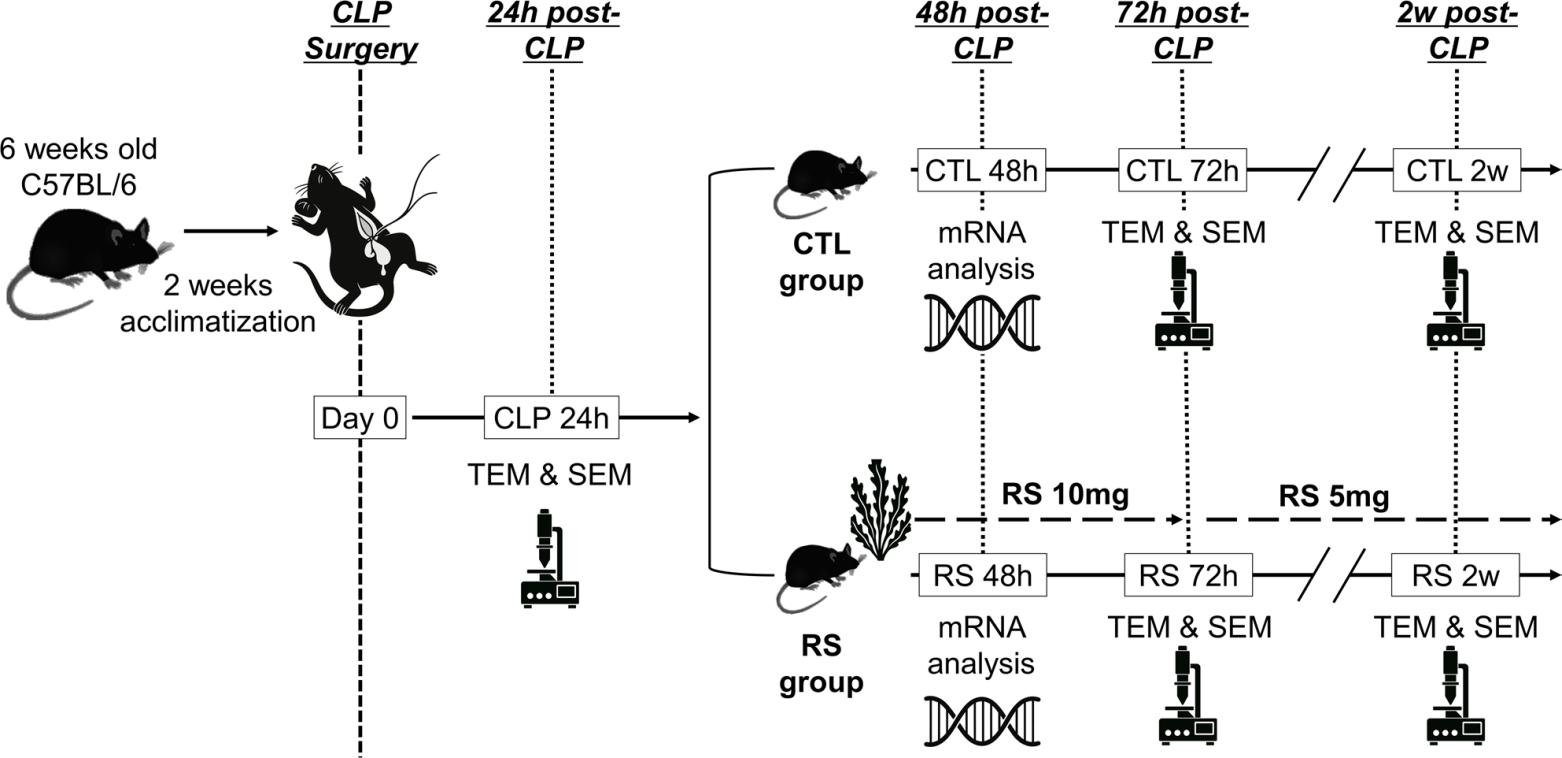
Experimental overview. CTL, control; RS, rhamnan sulfate; CLP, cecal ligation and puncture; TEM, transmission electron microscopy; SEM, scanning electron microscopy

**Fig. 2:**
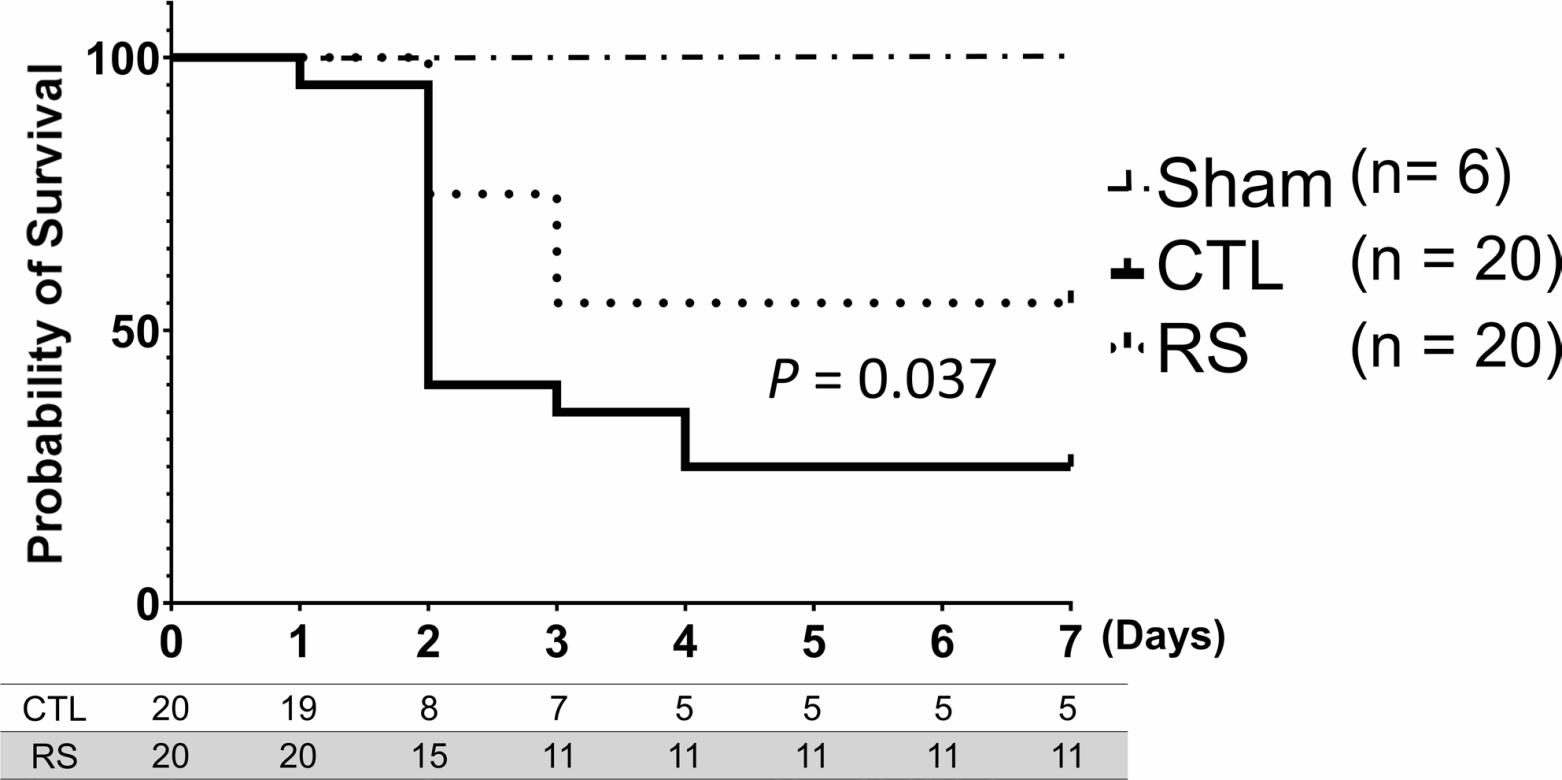
Kaplan-Meier survival curves. CTL, control; RS, rhamnan sulfate

Figure 1 shows an overview of the experiments. A total of 20 animals were included in each CLP group (CTL and RS) and 6 in the sham group, with no mortality observed in the sham group. The 7‑day survival rates were 55% (11/20) in the RS group and 25% (5/20) in the CTL group (*p* = 0.037). Figure 2 shows the Kaplan-Meier curves with the number at risk displayed below the plot.

### Ultrastructural analysis of endothelial glycocalyx repair

**Fig. 3:**
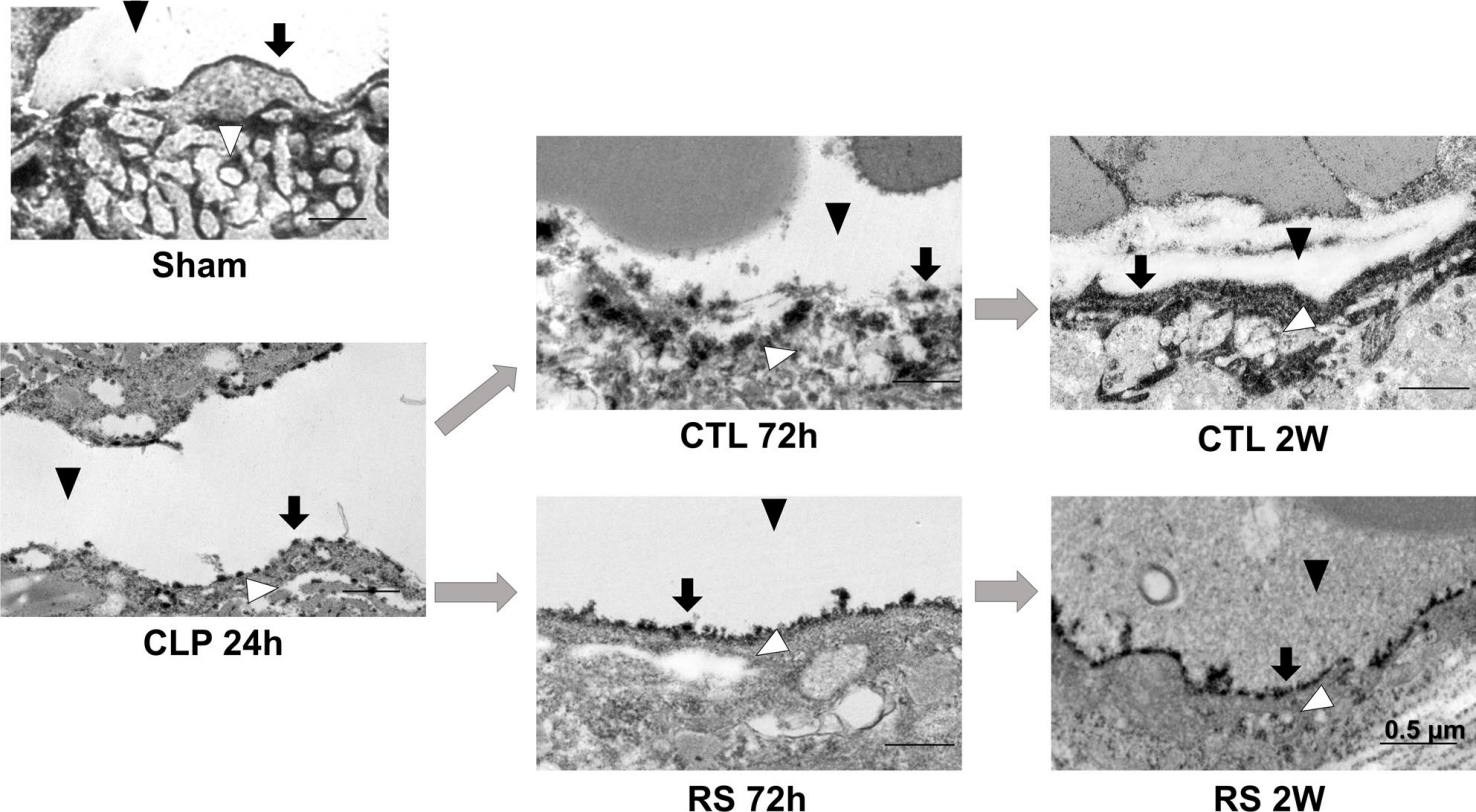
Transmission electron micrographs demonstrating the time course of ultrastructural changes during hepatic glycocalyx shedding. TEM images depicting the glycocalyx lining the hepatic microvasculature. The glycocalyx appears as a dark, electron-dense layer on the luminal surface due to lanthanum nitrate staining (black arrow). Black arrowheads indicate the sinusoidal lumen, and white arrowheads indicate the space of Disse. Bar = 0.5 μm in all images. CTL, control; RS, rhamnan sulfate; TEM, transmission electron microscopy

**Fig. 4:**
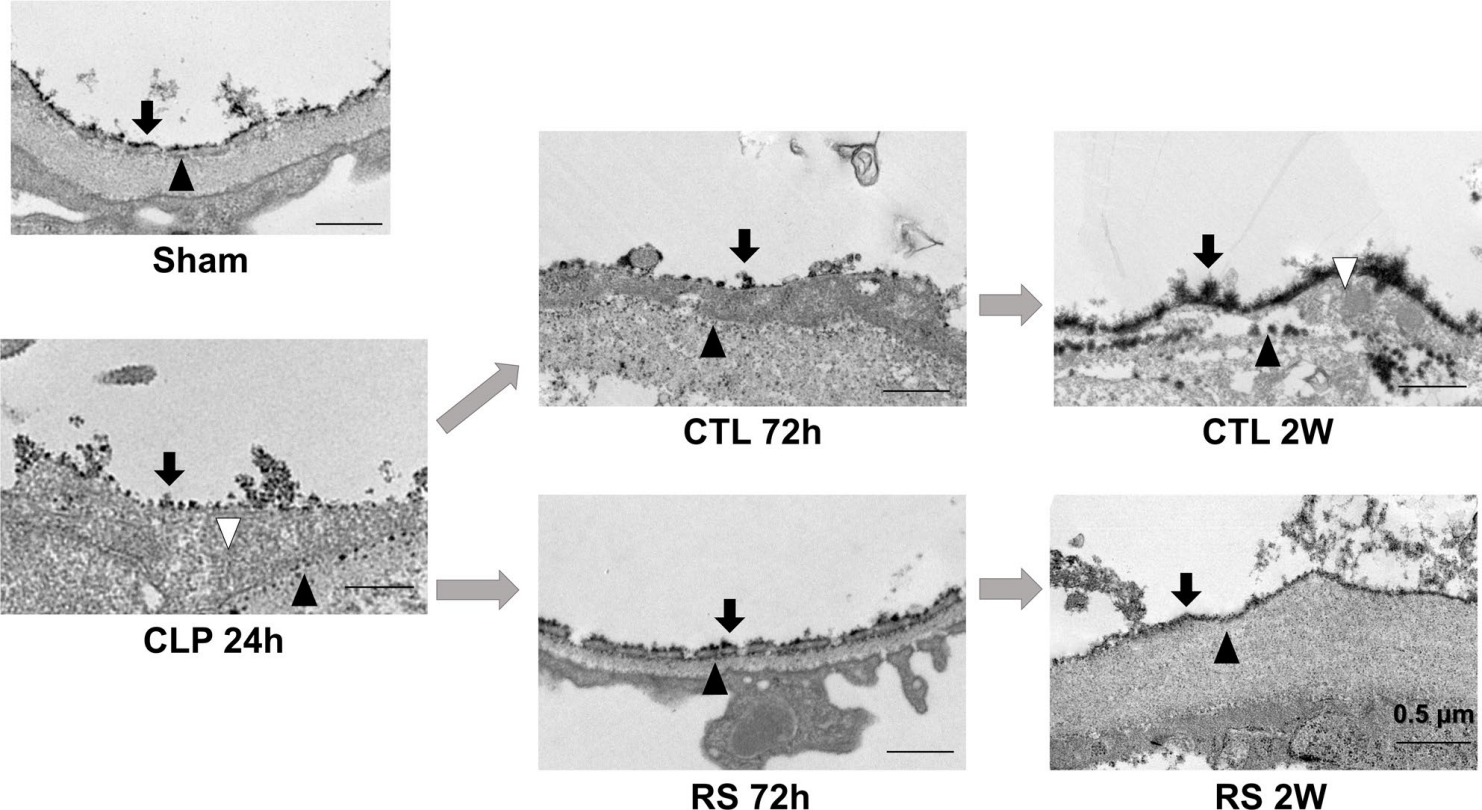
Transmission electron micrographs demonstrating the time course of ultrastructural changes during renal glycocalyx shedding. TEM images depicting the glycocalyx lining the renal microvasculature. The glycocalyx appears as a dark, electron-dense layer on the luminal surface due to lanthanum nitrate staining (black arrow). Black arrowheads indicate the glomerular basement membrane, and white arrowheads indicate the endothelial cell layer. Annotations are applied consistently across all panels. Bar = 0.5 μm in all images. CTL, control; RS, rhamnan sulfate; TEM, transmission electron microscopy

**Fig. 5:**
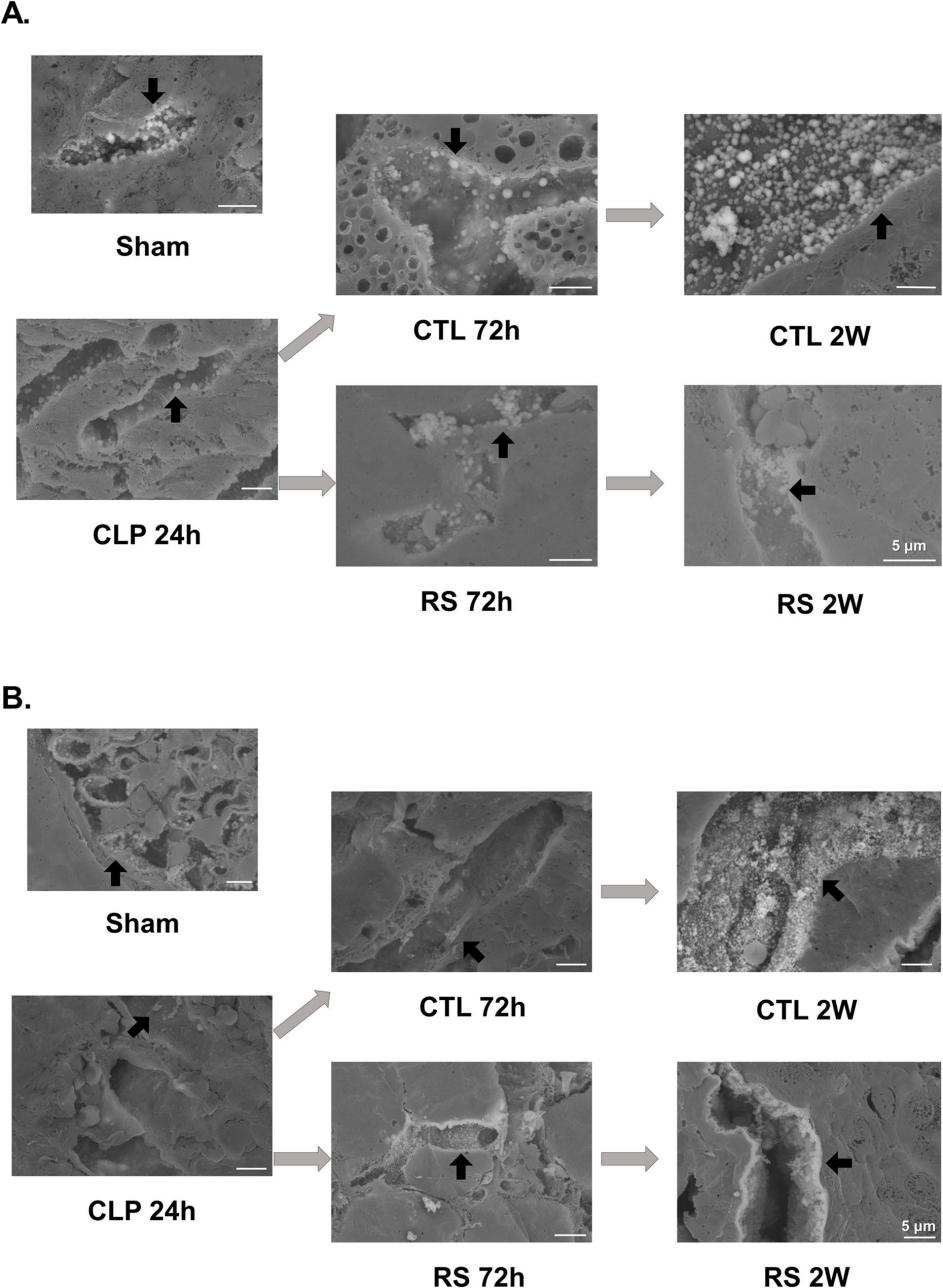
Scanning electron micrographs showing the glycocalyx in the liver and kidney. SEM images of the liver (**A**) and kidney (**B**). White granules indicate the glycocalyx stained with lanthanum nitrate (black arrow). Bar = 5 μm in all images. CTL, control; RS, rhamnan sulfate; SEM, scanning electron microscopy

To investigate the repair process of endothelial glycocalyx after sepsis, liver and kidney samples from the CTL and RS groups were observed by TEM and SEM. TEM images revealed the structure of the vascular endothelium layer (Figs. 3 and 4), and SEM images visualized the glycocalyx particles on the vascular endothelium (Fig. 5).

In the sham-treated mice, TEM images after lanthanum nitrate staining revealed a continuous, thick, mossy endothelial glycocalyx covering the endothelium of the liver (Fig. 3) and kidney capillaries (Fig. 4). SEM images also revealed a uniform distribution of glycocalyx particles covering the endothelial surface of the liver (Fig. 5A) and kidney (Fig. 5B).

At 24 h after CLP, TEM images in the CTL group (Figs. 3 and 4) showed that the endothelial cell glycocalyx was markedly disrupted and thinned compared to the sham-treated mice. The glycocalyx had lost its continuity, and surface coverage was significantly reduced. In some areas, the glycocalyx was observed as aggregated clumps. SEM images (Fig. 5) revealed denudation of the vascular endothelium with detachment of glycocalyx particles.

At 72 h after CLP, similar findings were observed in the CTL group. TEM continued to show loss of continuity (Figs. 3 and 4), and SEM demonstrated marked heterogeneity in glycocalyx particle size (Fig. 5). In the RS group, TEM images (Figs. 3 and 4) revealed that the endothelial glycocalyx had regained its continuity and was thicker than that at 24 h after CLP. SEM images (Fig. 5) showed a repopulation of glycocalyx particles covering the endothelial surface. Furthermore, the glycocalyx appeared to be composed of finer particles than in the CTL group. These findings were consistently observed in both the liver (Fig. 5A) and the kidney (Fig. 5B).

At two weeks after CLP, TEM images in the CTL group (Figs. 3 and 4) showed that the glycocalyx had regained continuity but appeared thicker than that observed in the sham-treated mice. SEM images (Fig. 5) showed that the particle morphology closely resembled that observed in the sham-treated group. In the RS group, TEM and SEM images (Figs. 3, 4 and 5) showed that the glycocalyx structure had normalized and was comparable to that observed in the sham-treated group.

Quantitative TEM analysis demonstrated marked structural alteration of endothelial glycocalyx after CLP (Supplementary Figs. 1 and 2). Although apparent glycocalyx thickness showed abnormal time-dependent changes after CLP, linear coverage and ultrastructural continuity were disrupted, suggesting glycocalyx injury with irregular aggregation and remodeling rather than restoration of abnormal glycocalyx layer. RS treatment significantly shifted glycocalyx thickness toward sham levels at 72 h and 2 weeks after CLP. Linear coverage did not show a statistically significant difference between the CLP and CLP + RS groups; however, RS treatment exhibited a trend toward improved continuity of the luminal glycocalyx layer.

### Quantitative real-time PCR (qPCR)

**Fig. 6:**
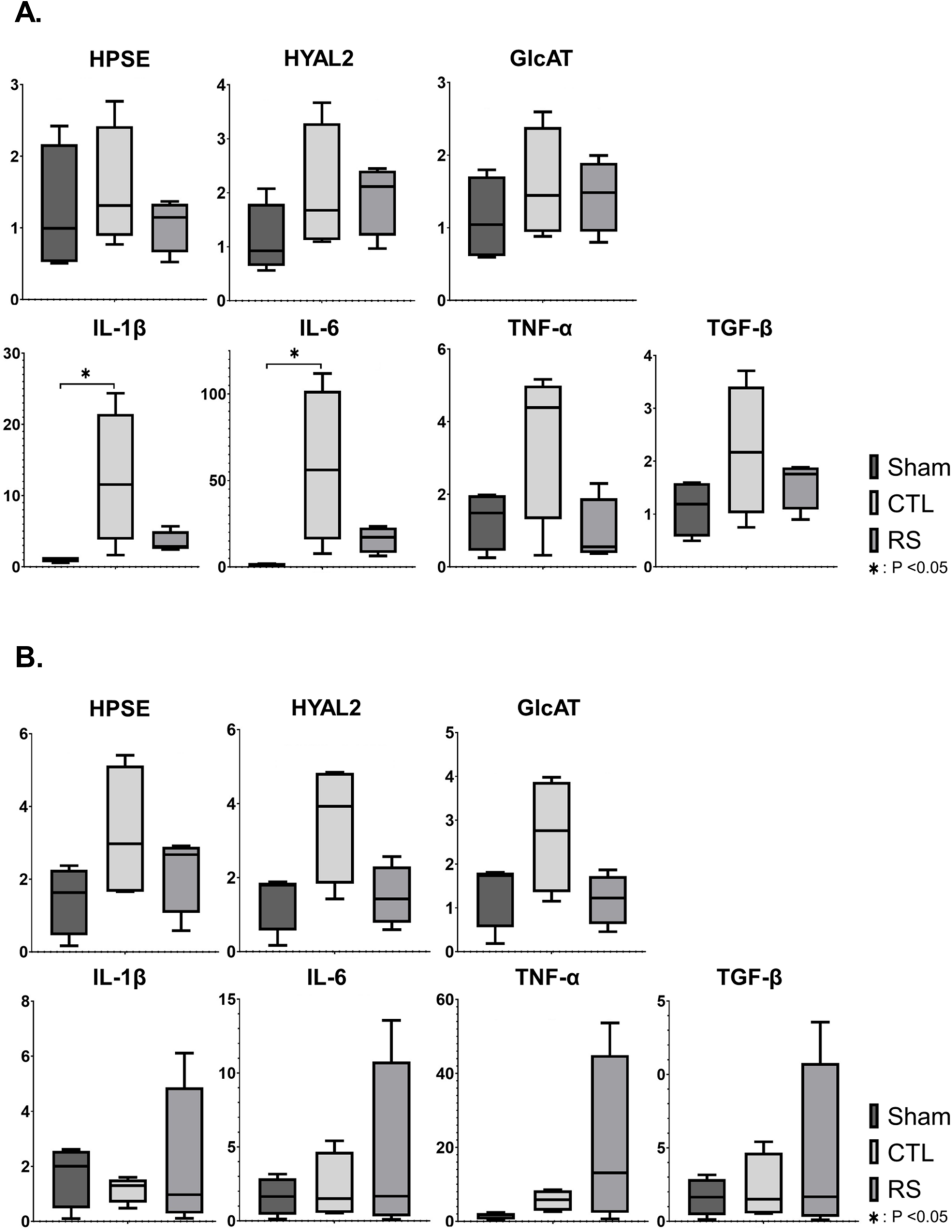
Box plots of the expression of genes related to glycocalyx metabolism and inflammation in the small intestine and the liver. Quantitative real-time PCR results of the small intestine (A) and liver (B). Box plots showing normalized complementary DNA expression levels of genes related to glycocalyx synthesis and degradation (heparanase [HPSE], hyaluronidase 2 [HYAL2], and glucuronyltransferase [GlcAT]) and inflammatory cytokines (interleukin-1β [IL-1β], interleukin-6 [IL-6], tumor necrosis factor-α [TNF-α], and transforming growth factor-β [TGF-β]) in the small intestine and liver. Data are presented as the median with interquartile range. An asterisk (*) indicates a statistically significant difference (*P* < 0.05). CTL, control; RS, rhamnan sulfate

qPCR revealed changes in the expression levels of genes related to glycocalyx degradation and inflammation among the groups (Fig. 6). In small intestine samples (Fig. 6A), the expression levels of HPSE and HYAL2, key genes involved in glycocalyx degradation, and the expression level of GlcAT, a key gene involved in glycocalyx biosynthesis, did not differ significantly among the groups. In particular, the expression levels of IL-1β and IL-6 in the CTL group were significantly elevated compared with the sham-treated group (*P* < 0.05). In contrast, the expression levels of IL-1β, IL-6, and TNF-α tended to be lower in the RS group than in the CTL group. In liver samples (Fig. 6B), the expression levels of HPSE and HYAL2 tended to be higher in the CTL group than in the sham-treated group and lower in the RS group than in the CTL group. No significant differences in GlcAT or inflammatory cytokine expression were observed among the groups.

### Plasma biomarker and cytokine measurements

Plasma syndecan‑1 and hyaluronan levels increased significantly after CLP, consistent with glycocalyx injury (Supplementary Fig. 3). Plasma cytokine levels, particularly IL-6 and TNF‑α, also increased after CLP, supporting successful induction of systemic inflammatory activation in this model (Supplementary Fig. 4). However, RS treatment did not significantly alter circulating Syndecan-1, HA, or cytokine levels at the analyzed time points. These findings indicate that the CLP procedure induced systemic endothelial injury and inflammatory activation, whereas the effects of RS were not clearly reflected in circulating biomarkers under the present experimental conditions.

## Discussion

In this study, we investigated the ultrastructural damage and repair process of the endothelial glycocalyx in a CLP-induced mouse sepsis model, focusing on the therapeutic effects of orally administered RS. Consistent with previous studies [8, 22, 23], our study also showed that 24 h after induction of sepsis, the endothelial glycocalyx in liver and kidney capillaries was thinned, had lost its continuity, and was fragmented into small particles, suggesting significant damage. These findings were observed by both TEM and SEM. The morphological changes persisted for at least 72 h after CLP, suggesting persisted endothelial damage and impaired repair of glycocalyx degradation. At two weeks after CLP, the glycocalyx in the CTL group appeared thicker than in the sham-treated group. This process indicates excessive repair and structural remodeling of the damaged glycocalyx. Notably, RS administration was associated with earlier and more robust repair of the endothelial glycocalyx. At 72 h after CLP, RS-treated mice displayed a continuous and thick glycocalyx layer compared with the CTL group, and at two weeks after CLP, they displayed ultrastructural features comparable to those of the sham-treated mice. SEM imaging further confirmed the regeneration of glycocalyx particles on the vascular surface of RS-treated mice.

Collectively, these findings indicate that RS may facilitate glycocalyx repair. The present results should be interpreted in the context of our previous work on orally administered RS [16, 18]. In a lipopolysaccharide‑induced model of acute inflammation, prophylactic RS reduced vascular leakage, organ injury, inflammatory cytokine expression, and preserved the pulmonary endothelial glycocalyx. In ApoE‑deficient mice, RS also ameliorated dyslipidemia and attenuated vascular inflammation, indicating vasculoprotective effects in chronic inflammatory settings. In contrast to these preventive and chronic‑phase studies, the present work was designed to determine whether RS can promote recovery after the onset of septic injury. Our findings suggest that post‑insult oral RS may support restoration and remodeling of the endothelial surface layer during sepsis recovery, extending the role of RS from preventing endothelial injury to facilitating glycocalyx repair after established systemic inflammation. Although partial spontaneous recovery was observed in the CTL group, the glycocalyx remained structurally abnormal, consistent with previous reports that regeneration under inflammatory conditions can be slow, heterogeneous, and prone to excessive or dysregulated remodeling. The endothelial glycocalyx is a dynamic structure involved in vascular permeability [24], leukocyte adhesion [25], and the regulation of blood coagulation [26, 27]. Glycocalyx degradation in sepsis is known to exacerbate endothelial dysfunction and organ damage. While spontaneous repair may occur over time, as evidenced by the partial recovery of glycocalyx observed in the CTL group, this process is likely slow and incomplete under septic conditions. In particular, the glycocalyx in the CTL group was observed to be thicker than that in the sham-treated group at two weeks, suggesting a state of excessive repair. Previous studies on glycocalyx damage caused by fluid shear stress have reported an increase in newly synthesized glycocalyx components and uniform remodeling during the regeneration process [28, 29]. This may suggest local thickening in the area affected by fluid shear stress. Furthermore, it is well known that the general tissue repair process progresses through the stages of hemostasis, inflammation, proliferation, and remodeling [30]. Our electron microscopy observations suggest that the repair process of the endothelial glycocalyx also progresses through a proliferation phase followed by remodeling, ultimately restoring the glycocalyx to its normal thickness. Further studies with more detailed time-course observations are needed. The accelerated recovery in RS-treated mice suggests that RS may promote glycocalyx regeneration by modulating the inflammatory response and inhibiting the activity of glycocalyx-degrading enzymes. Supporting this idea, qPCR analysis showed that the expression of inflammatory cytokines (IL-1β, IL-6, and TNF-α) in the small intestine tended to be lower in the RS-treated group than the CTL group. These results are consistent with previous reports demonstrating the anti-inflammatory effects of RS treatment [13, 16]. In the present study, RS suppressed inflammatory cytokine expression in the small intestine but not in the liver. This organ‑specific difference likely reflects the distinct biological roles of these tissues in CLP‑induced sepsis. The small intestine is the primary site of injury and inflammatory activation following CLP, and orally administered RS is expected to exert its strongest effects at this intestinal interface. In contrast, the liver predominantly reflects systemic inflammatory responses, and our additional cytokine analyses showed that RS did not attenuate systemic inflammation. These findings suggest that RS mainly modulates local intestinal inflammation rather than systemic cytokine responses. However, whether orally administered RS also exerts systemic effects requires further investigation.

The beneficial effects of RS against sepsis are thought to be mediated by modulating the intestinal microbiota, *Mucispirillum schaedleri*, after oral intake [31]. *M. schaedleri* has been reported to possess the ability to scavenge reactive oxygen species and molecular oxygen under inflammatory conditions, thereby mitigating oxidative stress-induced inflammation [31]. In that study, RS administration was associated with an increased abundance of *M. schaedleri*, which may have contributed to the suppression of intestinal inflammation. The early suppression of the inflammatory response in the RS group may have contributed to the prevention of persistent glycocalyx damage and the promotion of early repair. Furthermore, by controlling the proliferative phase, RS may also regulate the subsequent remodeling process. These molecular changes suggest that RS exerts its protective effects through a dual mechanism: inhibiting glycocalyx degradation and promoting biosynthetic pathways. While these findings suggest that RS modulates intestinal inflammation and contributes to glycocalyx preservation, the mechanism by which orally administered RS exerts its protective effects remains to be clarified. In this study, circulating RS levels were not measured, and systemic exposure could not be confirmed. However, partial intestinal uptake of sulfated polysaccharides is biologically plausible. Our previous work showed colocalization of RS with M cells in Peyer’s patches, and related compounds such as fucoidan have been detected in the systemic circulation after oral intake [32]. Thus, RS may act through local intestinal pathways, immune modulation, limited systemic absorption, or a combination of these mechanisms. Further pharmacokinetic studies are required to determine whether RS reaches plasma or tissues at biologically active levels.

In this study, the expression of glycocalyx-degrading enzymes (HPSE and HYAL2) tended to be suppressed in the RS-treated group compared with the CTL group. It has been reported that the expression of HPSE, which degrades heparan sulfate, and HYAL2, which degrades hyaluronic acid, is increased in sepsis [33–36]. Heparan sulfate is the main substrate of HPSE and a major component of syndecan-1, and it plays an important role in maintaining the integrity of the glycocalyx. Previous studies have shown that exogenous heparan sulfate and sphingosine-1-phosphate promote glycocalyx regeneration and restore inter-endothelial communication [37–39]. These findings imply that degradation of heparan sulfate by HPSE may significantly impair these functions, particularly as HPSE expression increases with the severity of sepsis and contributes to endothelial glycocalyx degradation [40]. This degradation promotes vascular permeability, leukocyte adhesion, and the release of pro-inflammatory cytokines, thereby affecting clinical outcomes [41].

Regarding HYAL2, its enzymatic activity leads to the generation of hyaluronic acid fragments with pro-inflammatory properties [35], suggesting that the increased HYAL2 expression may perpetuate the inflammatory cycle. Our findings suggest that RS administration suppressed HPSE and HYAL2 expression, which likely underlie both the attenuation of inflammation and the maintenance of the glycocalyx, as observed by the ultrastructural analysis. Importantly, the RS group had a higher survival rate than the CTL group, confirming the protective effect of RS suggested by the histological and molecular data. In this study, no significant differences were observed among the groups regarding the GlcAT gene involved in glycocalyx biosynthesis. Further investigation is warranted to identify key biosynthetic genes associated with glycocalyx regulation during sepsis. In addition, because only a single therapeutic dosing regimen was tested, the optimal dose and timing of RS administration remain to be clarified in future studies.

To date, although many pharmacological agents have been investigated for their potential to protect and promote repair of the endothelial glycocalyx [11, 12], none have yet reached clinical use. Based on our findings, RS, a natural food-derived compound, may offer a therapeutic strategy for endothelial glycocalyx preservation and regeneration in sepsis.

## Conclusions

This study demonstrated that the repair process of endothelial glycocalyx in septic mice can be evaluated by electron microscopy. Observations indicated that glycocalyx detachment showed little sign of repair after 72 h, but excessive remodeling was observed by day 14. Furthermore, RS may promote early endothelial glycocalyx repair and suppress excessive remodeling in the later stages. The observed anti-inflammatory effects were primarily limited to the intestine. These results may provide a basis for new therapeutic approach to maintaining vascular integrity in sepsis.

## Supplementary Information

Supplementary Material 1

## Data Availability

The datasets used and/or analyzed during the current study are available from the corresponding author on reasonable request.

## Abbreviations

RS: Rhamnan sulfate
GlcAT: Glucuronyltransferase
HPSE: Heparinase
HYAL2: Hyaluronidase
IL-1β: Interleukin-1β
IL-6: Interleukin-6
TNF-α: Tumor necrosis factor-α
TGF-β: Transforming growth factor-β
CLP: Cecal ligation and puncture
CTL: Control
SEM: Scanning electron microscopy
TEM: Transmission electron microscopy
