# Supplementary material for: Sepsis-induced endothelial glycocalyx disruption and remodeling: an ultrastructural study in mice
Source: Intensive Care Med Exp. 2026 Jul 20;14:98. doi: 10.1186/s40635-026-00954-w (PMC13385290; doi:10.1186/s40635-026-00954-w)

**Supplemental materials**

Sepsis-Induced Endothelial Glycocalyx Disruption and Remodeling: An Ultrastructural Study in Mice

Ryo Hisamune^1^, Kazuma Yamakawa^1^, Hong Wu^2^, Yoshihiko Fujioka^2^, Katsuhide Kayano^1^, Noritaka Ushio^1^, Rintaro Oide^3^, Masahiro Terasawa^4,5^, Koji Suzuki^4^, Takashi Nakano^2^, and Akira Takasu^1^

^1^ Department of Emergency and Critical Care Medicine, Osaka Medical and Pharmaceutical University, Takatsuki, Japan

^2^ Department of Microbiology and Infection Control, Faculty of Medicine, Osaka Medical and Pharmaceutical University, Takatsuki, Japan

^3^ Department of Translational Research, Osaka Medical and Pharmaceutical University, Takatsuki, Japan

^4^ Faculty of Pharmaceutical Sciences, Suzuka University of Medical Science, Suzuka, Japan

^5^ Konan Chemical Manufacturing, Co., Ltd., Yokkaichi, Japan

***Corresponding author:** Kazuma Yamakawa, MD, PhD

Department of Emergency and Critical Care Medicine, Osaka Medical and Pharmaceutical University, 2-7 Daigakumachi, Takatsuki, Osaka 569-8686, Japan.

Office: +81-72-683-1221; Fax: +81-72-684-6523

E-mail: [kazuma.yamakawa@ompu.ac.jp](mailto:kazuma.yamakawa@ompu.ac.jp)

**Supplementary Figure 1.** Quantification of glycocalyx thickness in transmission electron microscopy.

(A) Kidney. (B) Liver. Glycocalyx thickness was quantified from transmission electron microscopy images in each experimental group. Data are presented as box‑and‑whisker plots (median, interquartile range, and Tukey whiskers). Statistical analysis was performed using the Kruskal-Wallis test followed by Dunn’s multiple comparisons test. **** indicates P < 0.0001.

Abbreviations: CLP24H, 24 hours after cecal ligation and puncture; CLP48H, 48 hours after cecal ligation and puncture; CLP72H, 72 hours after cecal ligation and puncture; RS, rhamnan sulfate.


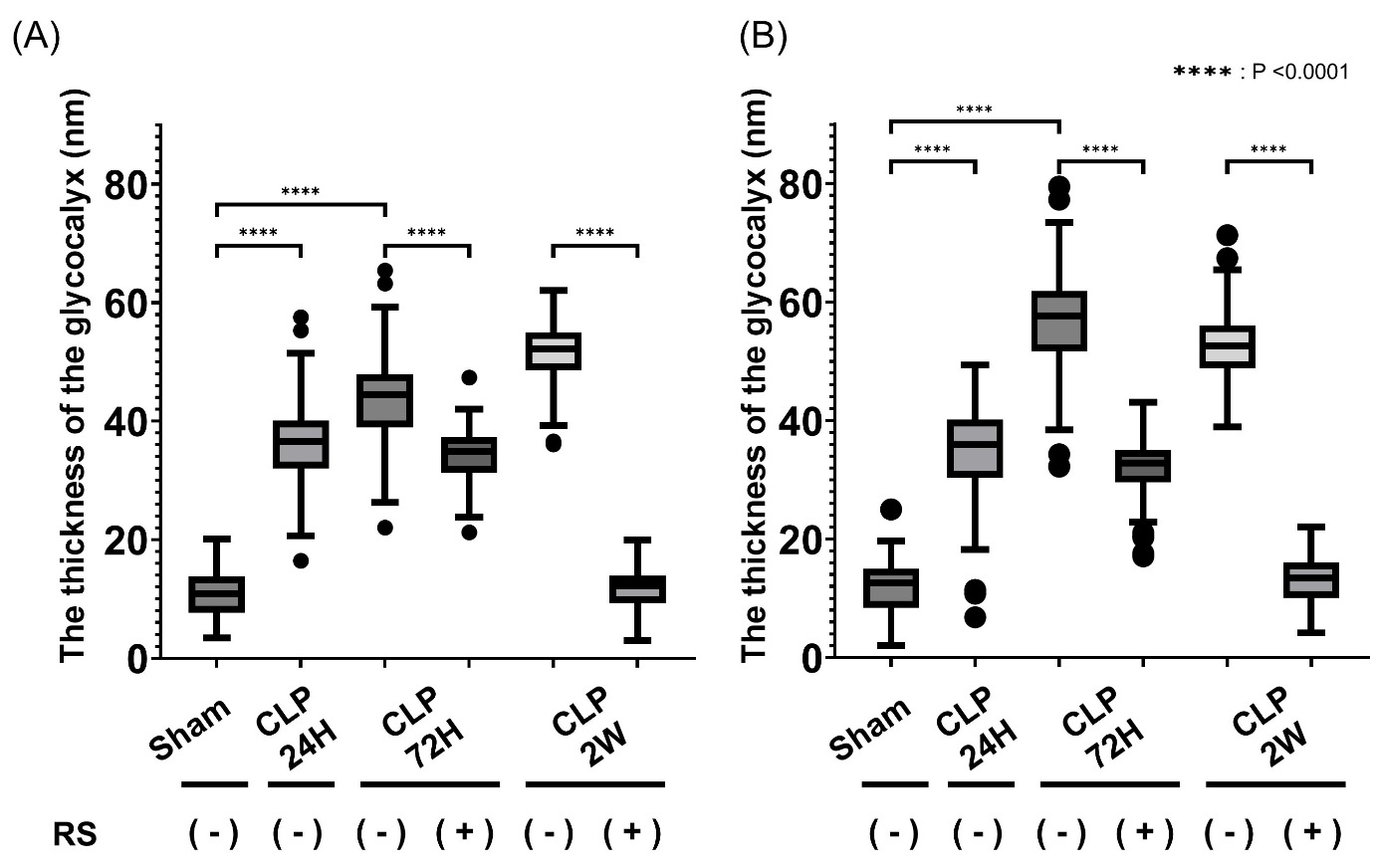


**Supplementary Figure 2.** Quantification of glycocalyx linear coverage in transmission electron microscopy.

(A) Kidney. (B) Liver. Glycocalyx linear coverage was quantified from transmission electron microscopy images in each experimental group. Data are presented as box‑and‑whisker plots (median, interquartile range, and Tukey whiskers). Statistical analysis was performed using the Kruskal-Wallis test followed by Dunn’s multiple comparisons test. * indicates P < 0.05; ** indicates P < 0.01.

Abbreviations: CLP24H, 24 hours after cecal ligation and puncture; CLP48H, 48 hours after cecal ligation and puncture; CLP72H, 72 hours after cecal ligation and puncture; ns, not significant; RS, rhamnan sulfate.


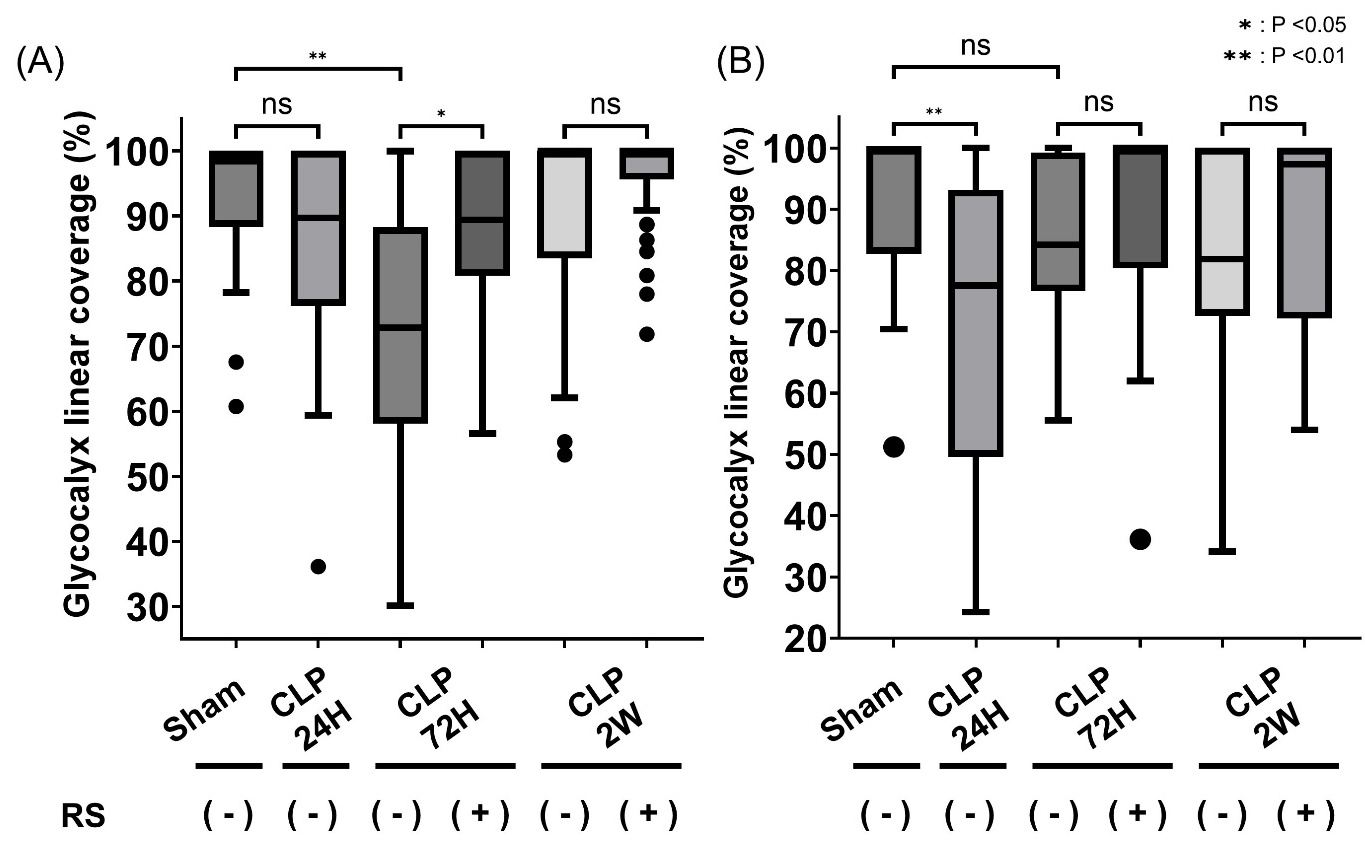


**Supplementary Figure 3.** Plasma levels of glycocalyx degradation markers.

(A) Syndecan‑1. (B) Hyaluronan. Plasma concentrations of glycocalyx degradation markers were measured in each experimental group. Data are presented as box‑and‑whisker plots (median, interquartile range, and Tukey whiskers). Statistical analysis was performed using the Kruskal-Wallis test followed by Dunn’s multiple comparisons test. * indicates P < 0.05; ** indicates P < 0.01.

Abbreviations: CLP24H, 24 hours after cecal ligation and puncture; CLP48H, 48 hours after cecal ligation and puncture; CLP72H, 72 hours after cecal ligation and puncture; ns, not significant; RS, rhamnan sulfate.


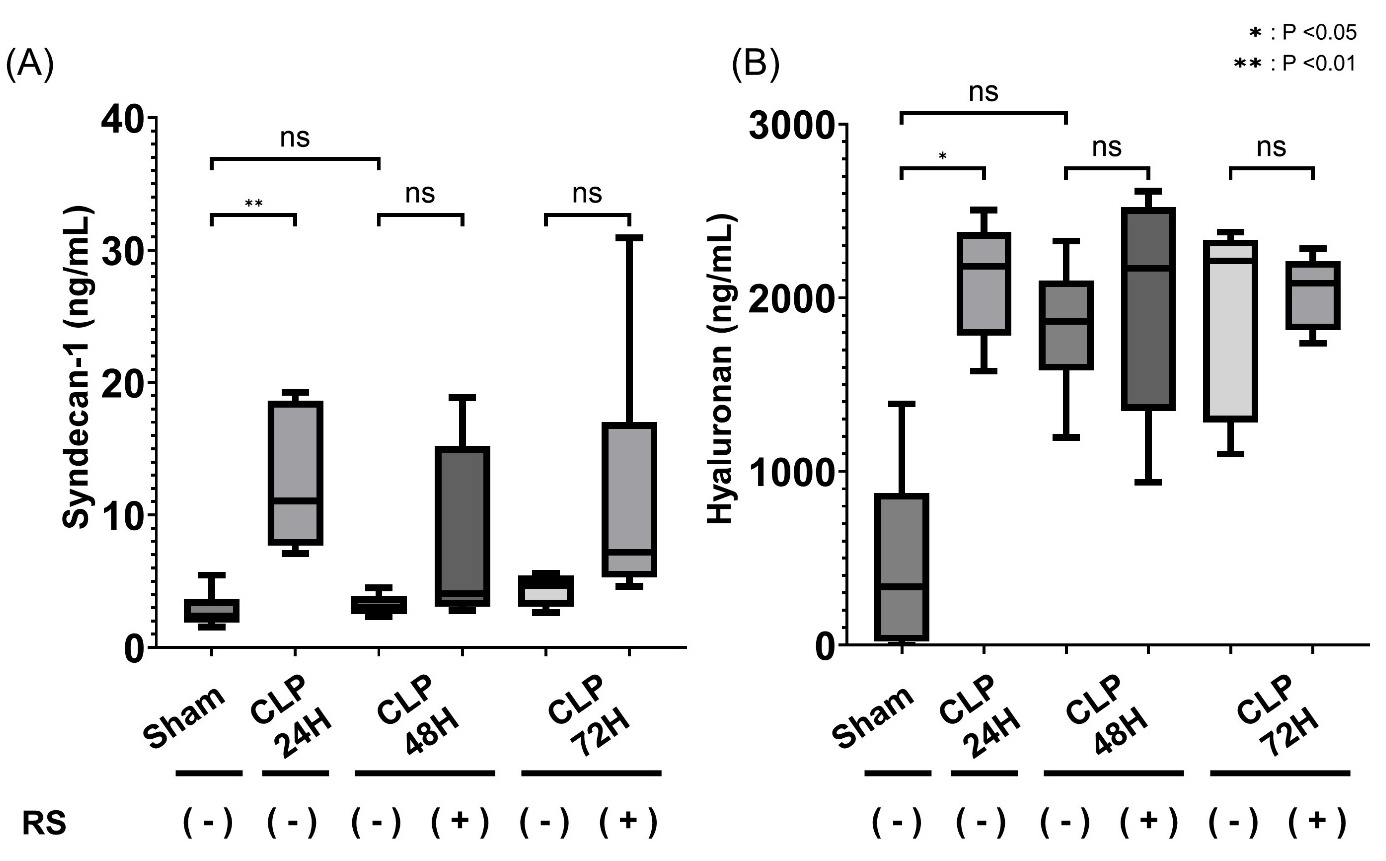


**Supplementary Figure 4.** Plasma cytokine levels measured by multiplex assay.

(A) IL‑1β. (B) IL‑6. (C) TNF‑α. Plasma cytokine concentrations were quantified using a multiplex immunoassay. The y‑axis for IL‑6 and TNF‑α is presented on a logarithmic scale. Data are shown as box‑and‑whisker plots (median, interquartile range, and Tukey whiskers). Statistical analysis was performed using the Kruskal-Wallis test followed by Dunn’s multiple comparisons test. * indicates P < 0.05; ** indicates P < 0.01; *** indicates P < 0.001.

Abbreviations: CLP24H, 24 hours after cecal ligation and puncture; CLP48H, 48 hours after cecal ligation and puncture; CLP72H, 72 hours after cecal ligation and puncture; IL, interleukin; ns, not significant; RS, rhamnan sulfate; TNF, tumor necrosis factor.


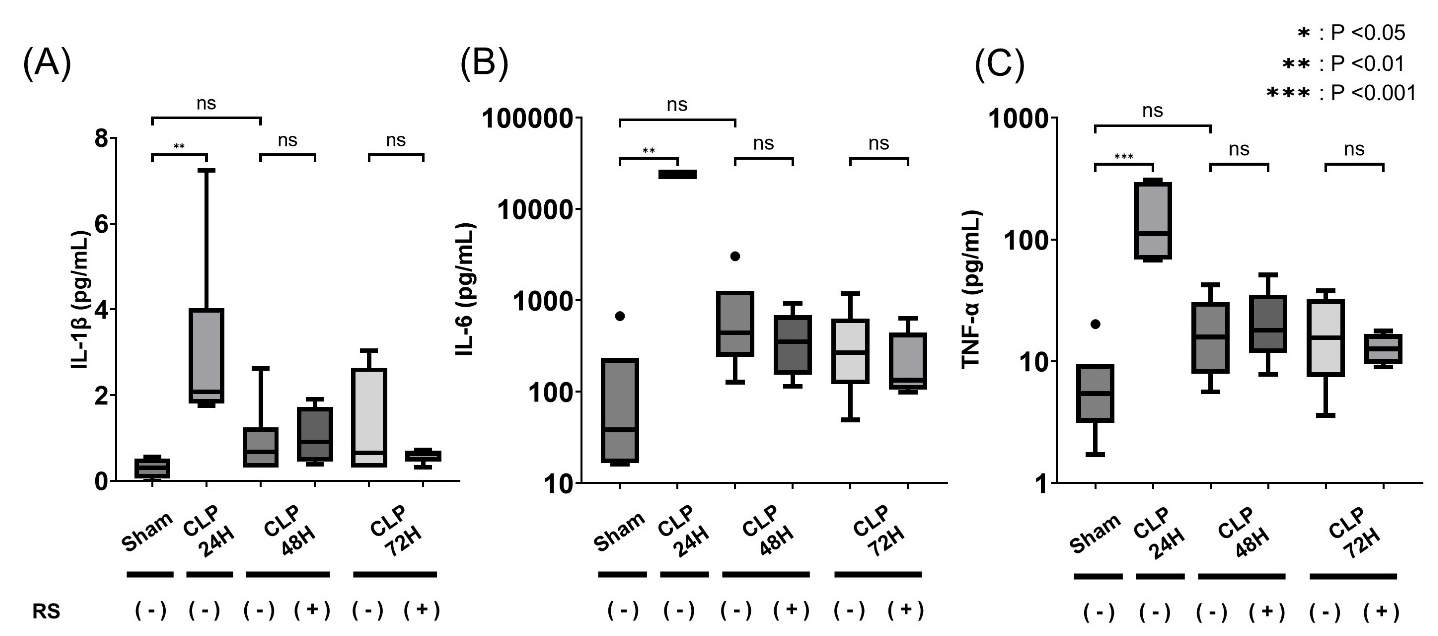

Supplement: Supplementary file 1 — Supplementary Material 1 [file 40635_2026_954_MOESM1_ESM.docx]
